# Ultra-Small Pd(0) Nanoparticles into a Designed Semisynthetic Lipase: An Efficient and Recyclable Heterogeneous Biohybrid Catalyst for the Heck Reaction under Mild Conditions

**DOI:** 10.3390/molecules23092358

**Published:** 2018-09-14

**Authors:** David Lopez-Tejedor, Blanca de las Rivas, Jose M. Palomo

**Affiliations:** 1 Department of Biocatalysis, Institute of Catalysis (CSIC), Marie Curie 2, Cantoblanco Campus UAM, 28049 Madrid, Spain; david.lopez@csic.es; 2 Institute of Food Science, Technology and Nutrition (ICTAN-CSIC), José Antonio Novais 10, 28040 Madrid, Spain; blanca.r@csic.es

**Keywords:** heterogeneous catalyst, palladium nanoparticles, semisynthetic enzyme, lipase, Heck reaction

## Abstract

A novel heterogeneous enzyme-palladium (Pd) (0) nanoparticles (PdNPs) bionanohybrid has been synthesized by an efficient, green, and straightforward methodology. A designed *Geobacillus thermocatenulatus* lipase (GTL) variant genetically and then chemically modified by the introduction of a tailor-made cysteine-containing complementary peptide- was used as the stabilizing and reducing agent for the in situ formation of ultra-small PdNPs nanoparticles embedded on the protein structure. This bionanohybrid was an excellent catalyst in the synthesis of *trans*-ethyl cinnamate by Heck reaction at 65 °C. It showed the best catalytic performance in dimethylformamide (DMF) containing 10–25% of water as a solvent but was also able to catalyze the reaction in pure DMF or with a higher amount of water as co-solvent. The recyclability and stability were excellent, maintaining more than 90% of catalytic activity after five cycles of use.

## 1. Introduction

**Figure 1:**
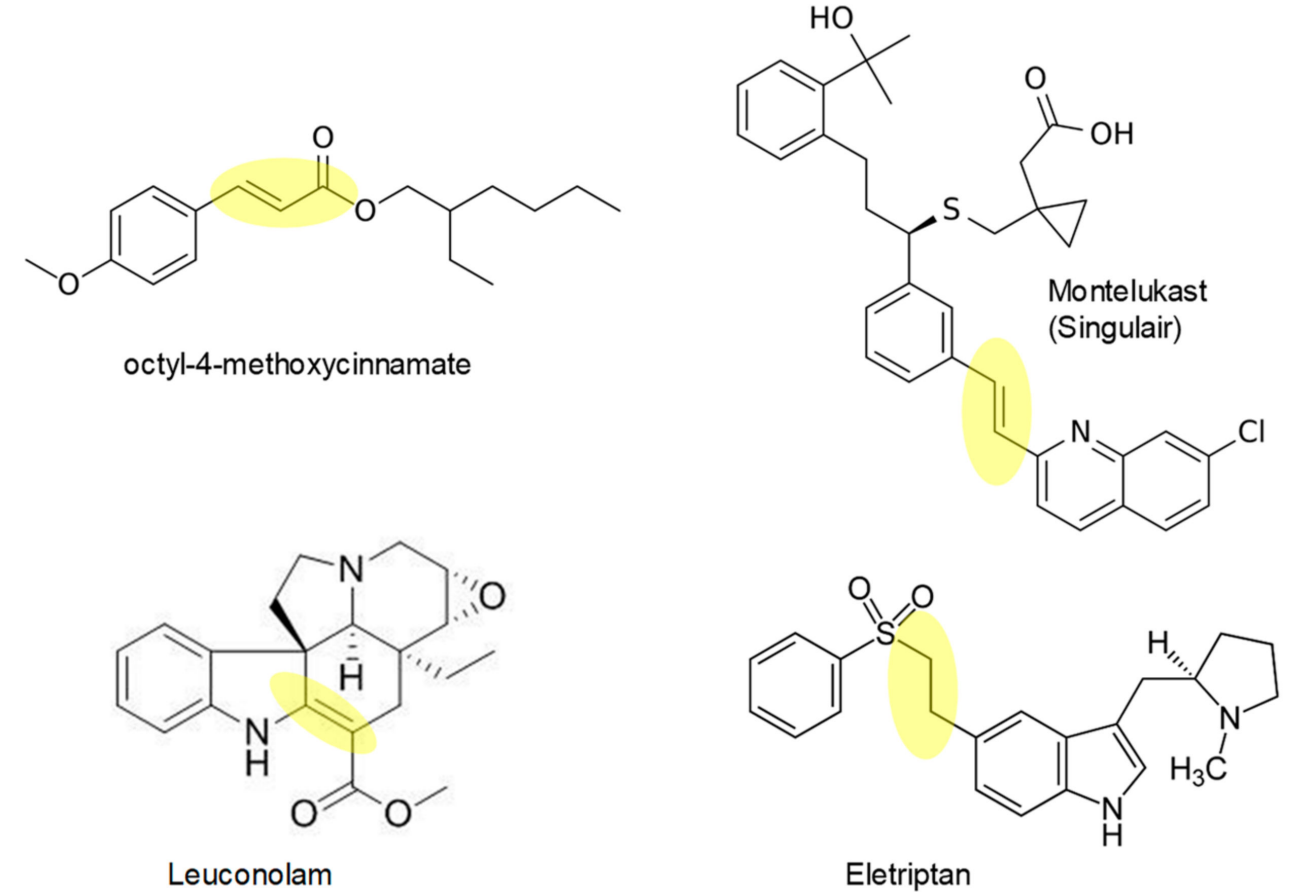
Pharmaceuticals synthesized by using Heck cross-coupling reaction.

The Heck reaction is one of the most studied coupling reactions for its usefulness, allowing the catalytic construction of C–C bonds under relatively mild conditions [1,2]. It has been widely used for the synthesis of a huge spectrum of molecules, natural products, polymers, and pharmaceuticals on both the laboratory and industrial scale. Some of the most notable products obtained using this method are Naproxen, Montelukast (Singulair), octyl-4-methoxycinnamate (present on UVB sunscreen), Electriptan or Leuconolam (Figure 1) [3,4].

Several metals and a huge range of ligands have been studied as catalysts for the reaction, with palladium (Pd) the preferred metal due to its tolerance to a wide variety of functional groups and ability to form C–C bonds between functionalized substrates, proceeding with stereo and regioselectivity and, usually, with good to excellent yields [2]. Investigation towards palladium-catalyzed Heck reaction has been focused on greener approaches to the reaction, performing it in aqueous media, and being able to recover and reuse the catalyst, which is quite important for industrial purposes [5,6,7,8].

In particular, the application of Pd nanoparticles as catalysts presents important advantages compared to other Pd molecules due to the high surface-to-volume ratio and very active surface atoms [9,10].

As described for other types of Pd complexes, optimization in the synthetic conditions to avoid hard or extreme conditions in the synthesis of Pd nanoparticles is greatly desirable. The application of biomolecules to induce the synthesis of these nanoparticles has been recently described [11]. The application of carbohydrates, peptides, polymers or microorganisms has been successfully applied [6,7,8,11,12,13,14]. However, in some cases, the generation of large size nanoparticles, not well dispersed or even a mixture in the nanoparticle morphology by using these biomolecules, have made the application of enzymes as a biological entity the best alternative to the ones mentioned before [11,15]. 

However, for possible industrial implementation of this type of catalyst, enzyme production is a key point. Although this can be obtained from commercial suppliers, it is important to have a good and consistent production source to obtain enough enzyme to be translated into high volumes of the final nanobiomaterials. In addition, enzymes with good stability might be interesting for application on reactions in harsh conditions, high temperatures or in the presence of organic solvents. 

In this work, we have synthesized a novel palladium (Pd) (0) nanoparticle (PdNPs)-enzyme nanobiohybrid using a tailor-made designed enzyme to induce the formation of ultra-small Pd NPs into the protein network.

Thermoalkalophilic lipase from *Geobacillus thermocatenulatus* (GTL) was genetically modified by introducing an unique cysteine (Cys) in a particular position and was overexpressed in *Eschericia coli*. The recombinant enzyme was then modified site-specifically by using a cysteine-containing peptide via disulfide exchange chemistry. This novel bionanohybrid was characterized by transmission electron microscopy (TEM) and X-ray diffraction (XRD) to confirm the existence of zero valent Pd and the final size and morphology of the nanoparticles. 

The potential synthetic application of this Pd-enzyme bionanohybrid as nanocatalyst was evaluated in Heck Cross-Coupling reaction under different conditions. Stability and reusability of the heterogeneous catalyst were also evaluated.

## 2. Results and Discussion

### 2.1. Synthesis and Characterization of the Bionanohybrid

**Figure 2:**
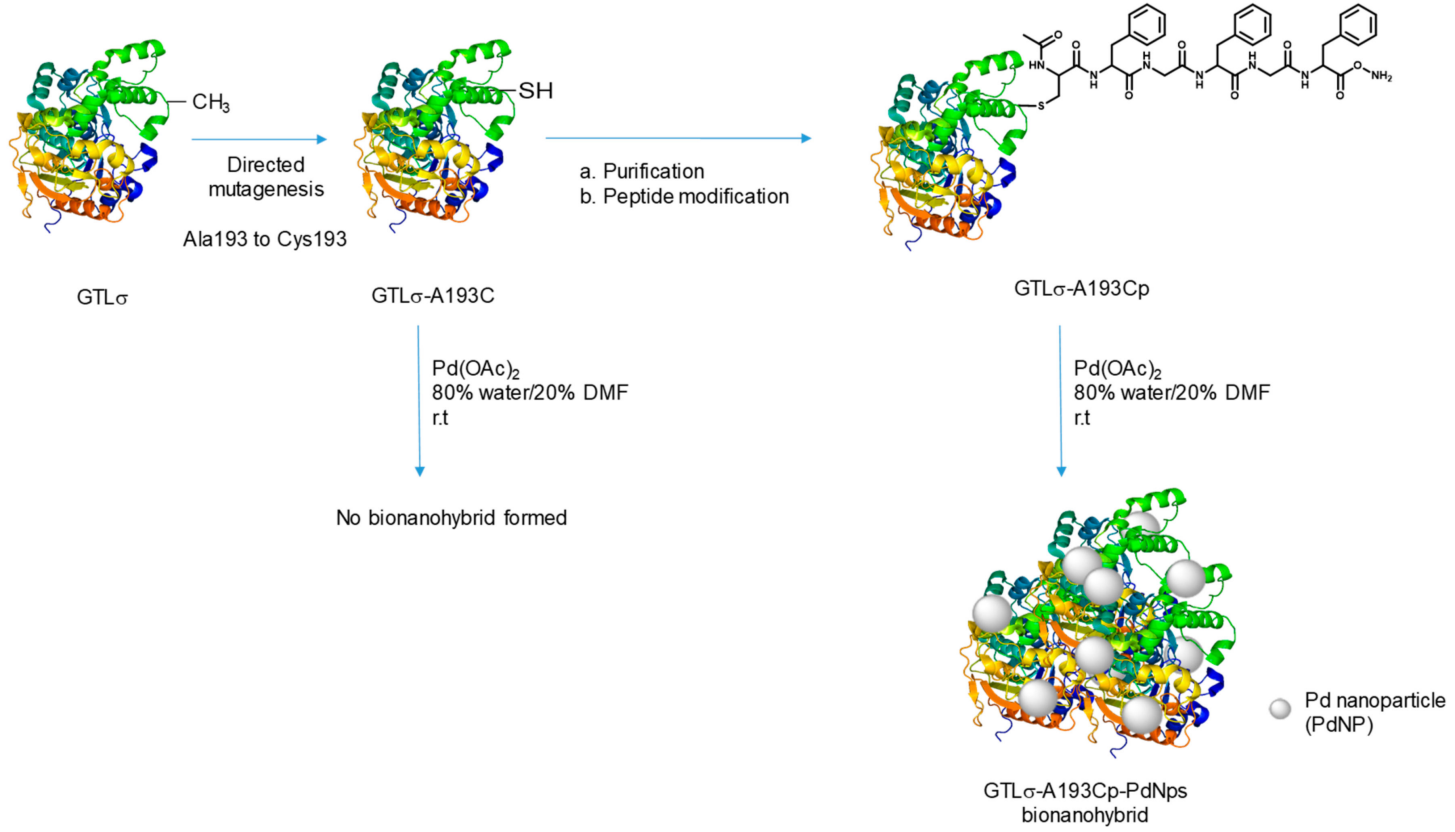
Preparation of the *Geobacillus thermocatenulatus* (GTL)σ-A193Cp- palladium (0) nanoparticles (PdNPs) bionanohybrid.

First, a tailor-made enzyme was created following the protocol previously described [16,17]. Lipase from *Geobacillus thermocatenulatus* (GTL) was genetically modified by site-directed mutagenesis, changing Ala193 to Cys (Figure 2), without alteration in the properties of the enzyme. This GTLσ-A193C variant was overexpressed in *Escherichia coli* and purified, which permitted to obtain a high amount of protein. 

Then, this new enzyme solution was combined with palladium acetate to create a new kind of bionanohybrid; nanocatalysts formed by in situ synthesized palladium nanoparticles in the protein network [18]. The formation of the bionanohybrid can be easily recognized by the increase in turbidity of the solution, which is precipitated after centrifugation.

In this case, no aggregate was obtained when the GTLσ-A193C and palladium salt mixture was incubated for 24 h at room temperature, with the solution remaining completely transparent. This result indicated that no bionanohybrid could be formed when using this genetically modified protein.

Therefore, this GTLσ-A193C was site-specific chemically modified at the cysteine by a Cys-containing peptide (Ac-Cys(PheGly)_2_PheCONH_2_) (Figure 2). First, the cysteine on the protein (C193) was protected with 2-dipyridiyldisulfide before the reaction. Then the peptide was conjugated to the Cys193 by a disulfide bond exchange. This modification generated a new semisynthetic GTL lipase (GTLσ-A193Cp) with improved properties and specifically increased the hydrophobicity of the final protein structure [16]. 

**Figure 3:**
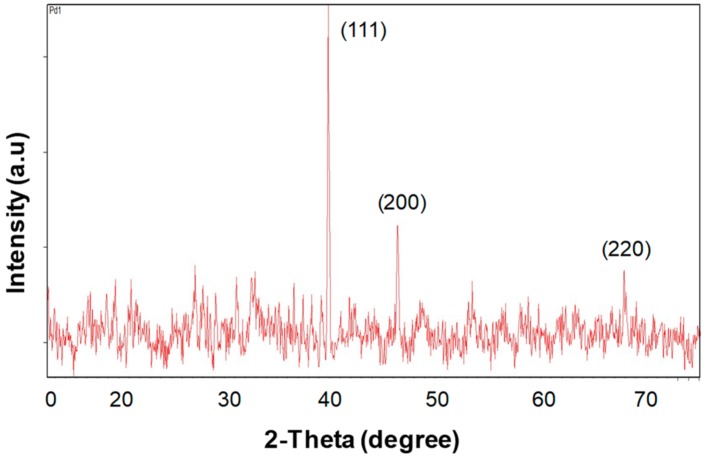
X-ray diffraction (XRD) pattern of GTLσ-A193Cp-PdNPs bionanohybrid.

Thus, the protocol for the bionanohybrid synthesis was applied using this new enzyme GTLσ-A193Cp. In this case, the solution turned cloudy in the first hour of incubation and finally aggregate was obtained after 24 h (Figure 2). The solid was recovered by centrifugation, washed and lyophilized. X-ray diffraction (XRD) analysis showed that zero-valent Pd was the only species present in the bionanohybrid (Figure 3). 

**Figure 4:**
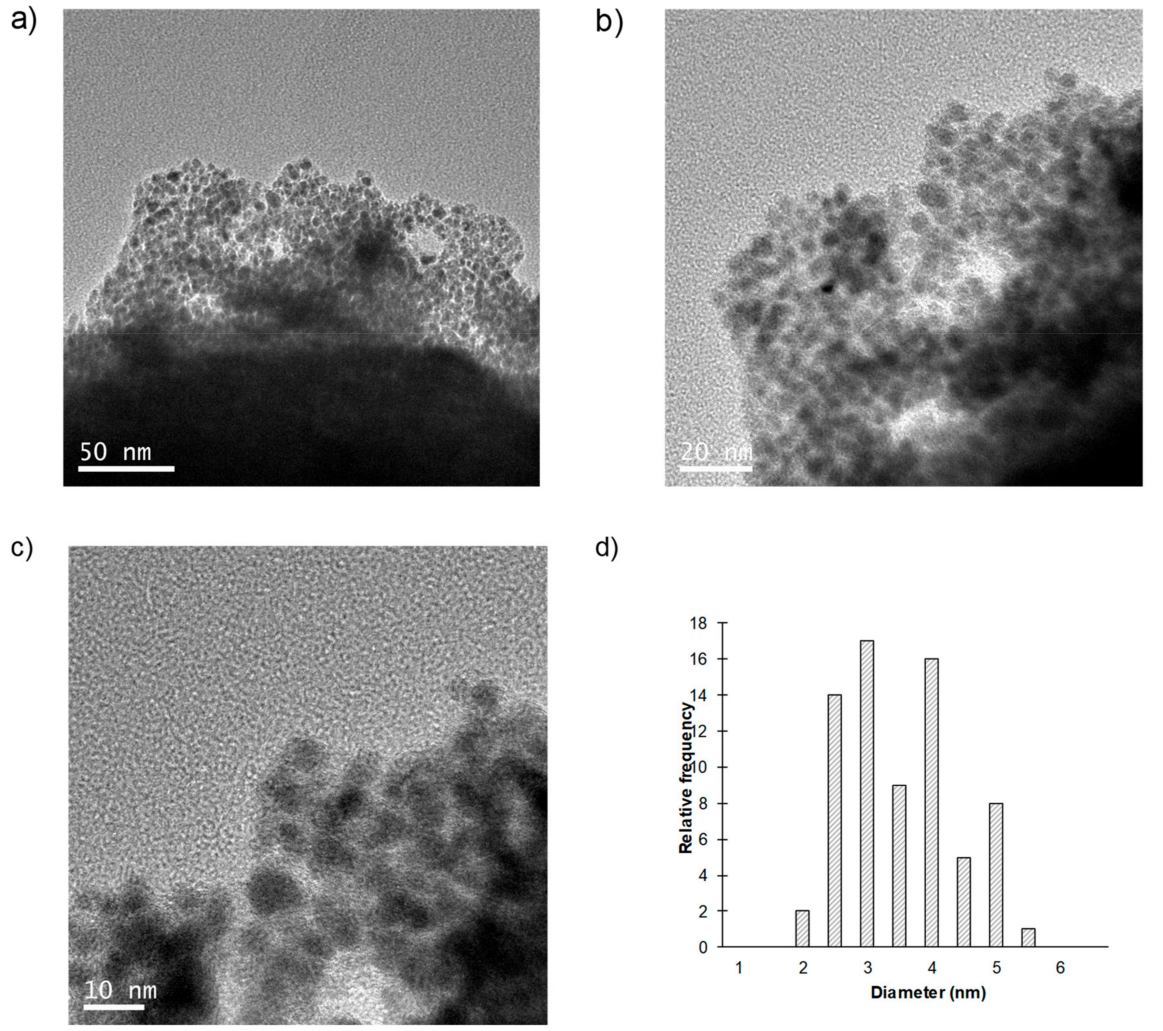
Characterization of GTLσ-A193Cp-PdNPs bionanohybrid. (**a**–**c**) transmission electron microscopy (TEM) and high resolution TEM (HRTEM) micrographs. (**d**) Pd NPs size distribution.

The content of palladium in the solid was 40 wt.% measured by inductively coupled plasma atomic emission spectrometry (ICP-OES). More than 99% of the enzyme offered was remaining in the final bionanohybrid confirmed by Bradford assay [19]. TEM analysis showed that ultra-small spherical palladium nanoparticles (PdNPs) were synthesized into the protein network of GTLσ-A193Cp with a size distribution of 2 to 4 nm, but mainly with a size around 3 nm (Figure 4).

The proposed mechanism for the formation of this enzyme-metal nanobiohybrid consists in two different steps, (i) The ionic binding of the Pd^2+^ ions with different aminoacid residues of the protein, and (ii) the nucleation, reduction of Pd^2+^ to Pd(0) with the final growth of the nanoparticle. One of the important points in this mechanism is the role of aminoacids and small peptide sequences into the protein involved during the nanobiohybrid formation. The phenomenon that peptides sequences can be reduced metal ions has been well studied using tailor-made synthetic small peptides. Therefore, the protein in question must contain particular peptides sequences, the so-called ideal peptides, which has to contain amino acids with moderate binding affinity for both metal ions and formed metal particles (i.e., amino acids presenting hydrophobic or charged side chains (with the charge sign opposite to that showed by the metal ions)) together with neighboring amino acids showing a strong reducing ability (i.e., especially amino acids presenting hydrophobic side chains, or particularly alcohol residues such as serines, threonines or tyrosines) [18].

Thus, adding the metal salt to the enzyme solutions, due to the binding ability of the identified sequences, rapid adsorption of soluble Pd ions on the enzyme surface was observed. Furthermore, the adsorbed metal ions act as a cross-linker between the enzyme’s molecules, reducing their solubility and finally inducing the initial fast precipitation. When the metal ions are adsorbed, the amino acids in protein surrounding them caused the nucleation.

**Figure 5:**
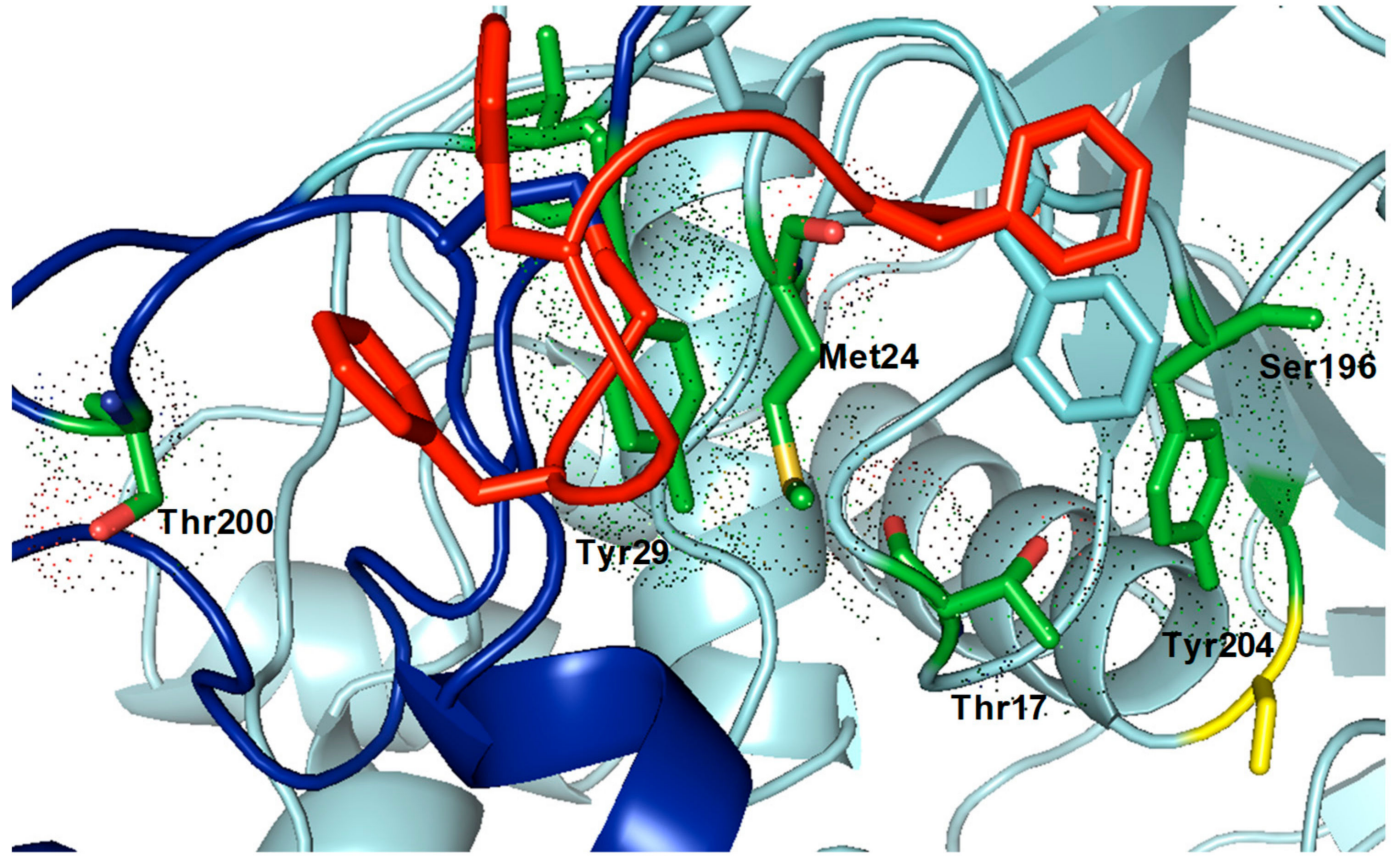
Crystal structure of the active form of the artificial enzyme GTLσ-A193Cp. Cartoon of the peptide sequence and the selected alcoholic protein residues. A193C-catalytic Ser114 is marked in yellow, lid in blue, peptide in red and the different Ser, Thr, Tyr or Met residues in green. All pictures were created by using Pymol.

The structural analysis of the chemically modified variant demonstrated that the site-specific location of the hydrophobic peptide (Figure 5) was critical, considering the necessity of hydrophilic groups together to hydrophobic ones for the reducing step. Serine, threonine, tyrosine or even methionine were surrounded by the peptide (Figure 5). These groups were able to reduce the adsorbed Pd^2+^ to Pd(0), which is mandatory for the nucleation process [18].

In contrast, the native variant without the peptide also presented a hydrophobic area on the lid side (Figure S1, see Supplementary Materials), and although it could be possible from the initial adsorption of Pd^2+^, no adequate hydrophilic groups were surrounding that position which could demonstrate why the native variant was unable to form the bionanohybrid.

### 2.2. Heck Reaction

**Scheme 1:**
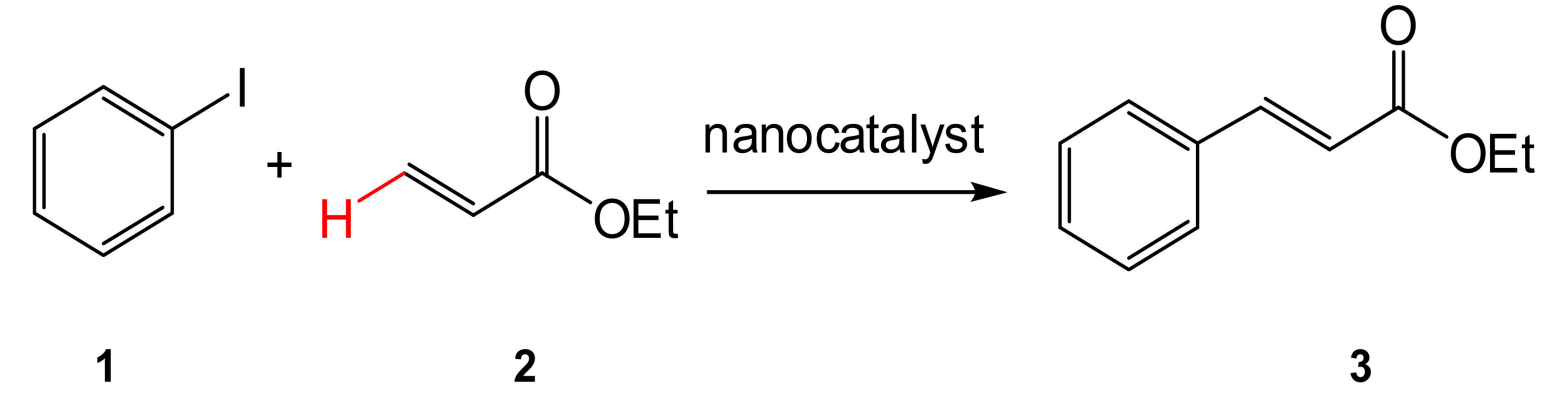
C–C Heck reaction catalyzed by bionanohybrid.

Thus, this new GTLσ-A193Cp-PdNPs bionanohybrid was tested as a heterogeneous catalyst in Heck reaction using iodobenzene (**1**) and ethyl acrylate (**2**) as substrates (Scheme 1). The reaction was performed at moderate conditions, in an aqueous solution, containing 75% (*v*/*v*) dimethylformamide (DMF) and 65 °C.

The C–C reaction was evaluated using different bases in the reaction (Table 1). The application of dimethylaminopyridine (DMAP) as the base resulted in no conversion (Table 1, entry 1). The nanocatalyst exhibited around 20% conversion after 24 h when the combination of DMAP and triethylamine was used (Table 1, entry 2). The best result was obtained using triethylamine, where 78% of ethyl cinnamate (**3**) was obtained with a TOF value of 2.96 h^−1^, more than three times compared with the use of DMAP and with a complete reaction (>99%) at 48 h (Table 1, entry 3). In addition, diisopropylethylamine (DIPEA) was evaluated, although the achieved conversion was slightly lower after 24 h compared to the one with triethylamine (Table 1) with a TOF value of 2.32 h^−1^. In all cases where a reaction was obtained, a high selectivity was achieved, producing the *trans*-isomer exclusively.

The increase in the amount of triethylamine in the reaction catalyzed by GTLσ-A193Cp-PdNPs bionanohybrid was studied. This addition caused a slight negative effect in the final conversion value, with 5 eq obtaining 41% of **3**, but especially after 10 eq, where only 7% of **3** was achieved after 24 h (Table 1, entries 5 and 6), a TOF value of 0.27 h^−1^, 15 times lower compared when 1.5 equivalent was used. The increase of **2** also showed a negative effect on the final conversion value (Table 1, entry 7).

**Figure 6:**
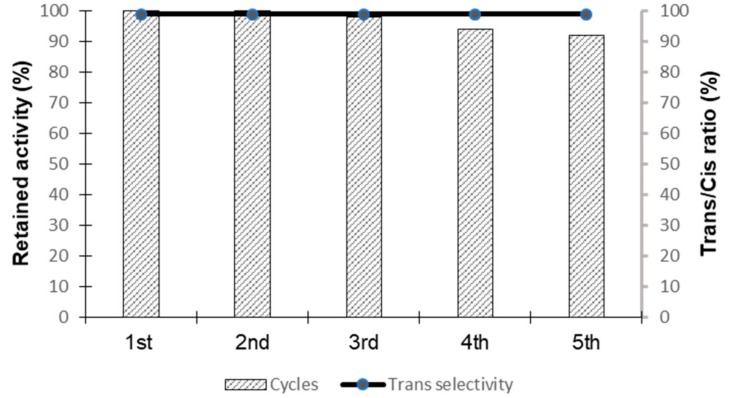
Recyclability of GTLσ-A193Cp-PdNPs bionanohybrid in the Heck reaction. Conditions: **1** (0.274 mmol), **2** (0.55 mmol), 1 mL DMF/H_2_O (75/25), 1.5 equiv NEt_3_, 1 mg of nanocatalyst, 65 °C.

For a possible industrial application, the recyclability of the heterogeneous nanocatalyst was studied. The GTLσ-A193Cp-PdNPs bionanohybrid was used in at least five cycles of Heck reaction at 25% water in DMF at 65 °C. The nanocatalysts kept 90–95% of the initial activity while also maintaining a complete *trans* selectivity (Figure 6).

To evaluate the capacity of this nanocatalyst, the Heck reaction was performed changing the volume of water in the reaction medium. The reduction in the volume of water in the medium was first attempted. Surprisingly, the bionanohybrid catalyzed the reaction faster when only 10% water was used, obtaining 86% conversion of **3** after 24 h, with a TOF value of 3.27 h^−1^ (Table 2, entry 2).

A very interesting result was also obtained when the reaction was performed in pure DMF as the solvent. The GTLσ-A193Cp-PdNPs bionanohybrid catalyzed the Heck reaction with 56% conversion after 24 h, TOF value of 2.13 h^−1^, with exclusively *trans* selectivity (Table 2, entry 1). This differed from the results previously obtained using bionanohybrid synthesized using other lipase where no conversion was achieved even at higher temperatures (Table 2, entry 5). This phenomenon could demonstrate that the application of a highly stable protein variant of GTL allows broader applicability of the final Pd NPs biohybrid in synthetic chemistry.

This argument was also corroborated when higher volumes of water were added to DMF as the solvent. This bionanohybrid also catalyzed the Heck reaction in the presence of 40% of water in DMF at 65 °C, with a TOF value of 1.90 h^−1^ (Table 2, entry 4), better than the previous comparison (Table 2, entry 7). In addition, in all cases, the *trans*-selectivity was conserved.

To demonstrate the broad scope of applicability of this new bionanohybrid, Suzuki cross-coupling reaction between bromobenzene and phenylboronic acid in aqueous media at mild conditions reaction in pure water was attempted (Table S1, see Supplementary Materials). The GTLσ-A193Cp-PdNPs showed good performance producing 70% conversion of biphenyl in 24 h at 50 °C in aqueous media, with a TOF value of 0.43 (Table S1). Furthermore, Sonogashira reaction between propargyl alcohol and iodoanisole was performed (Table S2). 

## 3. Materials and Methods 

### 3.1 General

Butyl-Sepharose^®^ 4 Fast Flow from GE Healthcare (Uppsala, Sweden), 2-dipyridyldisulfide (2-PDS), dithiothreitol (DTT), sodium borohydride, dimethylsulfoxide (DMSO), dimethylformamide (DMF), dimethylaminopyridine (DMAP), diisopropylethylamine (DIPEA), p-nitrophenylpropionate (pNPP), Pd(OAc)_2_, ethyl-acrylate, iodobenzene, *cis* and *trans* ethyl cinnamate, and triethylamine were from Sigma (St. Louis, MO, USA). Sucrose monolaurate was from Mitsubishi-Kagaku (Tokyo, Japan). AcN-Cys(SH)-Phe-Gly-Phe-Gly-Phe-CONH_2_ was purchased from Isogen (De Meern, Netherlands). Inductively coupled plasma atomic emission spectrometry (ICP-OES) of the acidic digestion of the solid powder of bionanohybrid was performed on a Perkin Elmer OPTIMA 2100 DV equipment. The transmission electron microscopy (TEM), high resolution TEM microscopy (HRTEM) were performed on a JEOL 2100F microscope equipped with an EDX detector INCA x-sight (Oxford Instruments, Tokyo, Japan). The X-ray diffraction (XRD) pattern was obtained using a Texture Analysis Diffractometer D8 Advance (Bruker) with Cu Kα radiation. 

### 3.2. Site-Directed Mutagenesis, Cloning, and Expression of Geobacillus Thermocatenulatus Lipase (GTL)

All site-directed mutagenesis experiments were carried out by polymerase chain reaction (PCR) using mutagenic primers. To introduce the amino acid change, the corresponding pair of primers was used as a homologous primer pair in a PCR reaction using a specific plasmid as template and Prime Start HS Takara DNA polymerase. The product of the PCR was digested with endonuclease DpnI that exclusively restricts methylated DNA [20]. *E. coli* DH10B cells were transformed directly with the digested product. The plasmid with mutated GTL was identified by sequencing and then transformed into *E. coli* BL21 (DE3) cells to express the corresponding proteins. First, C65S was created, and the resulting plasmid was used as a template to create the double mutant C65S/C296S-GTL (GTLσ). This plasmid (pT1BGTLmutCys) was used as a template to construct the additional mutation (A193C), creating the mutant GTLσ-A193C using different mutagenic primers: A193C (Ala/cys 193-5,5′-GAAAGCGtgcGCTGTCGCCAG; Ala/cys 193-3 5′-CTGGCGACAGCg caCGCTTTC). The gene corresponding to the mature lipase from *G. thermocatenulatus* (GTL) was cloned into a pT1 expression vector as previously described in Reference [20]. Cells carrying the recombinant plasmid pT1GTL were grown at 30 °C and over expression was induced by raising the temperature to 42 °C for 20 h. 

### 3.3. Purification of GTLσ-A193C

The crude extract from *E. coli* containing the GTL variant was diluted with 10 mM sodium phosphate at pH 7.0 to a concentration of 0.5 mg/mL. Then, butyl-Sepharose was added in a 1/20 (*v*/*v*) proportion and gently stirred overnight at 25 °C. Periodically, the activity of suspensions and supernatants were measured by the pNPP assay described above. After that, the adsorbed lipase preparation was abundantly washed with distilled water. Purification overall yield was 80% confirmed by sodium dodecyl sulfate polyacrylamide gel electrophoresis (SDS-PAGE) analysis and a decrease in the supernatant activity.

### 3.4. Irreversible Site-Specific Chemical Modification of GTLσ-A193C by Thiol-Containing Peptides

GTLσ-A193C adsorbed on butyl-Sepharose (0.2 g) was incubated in 2 mL of DTT solution (50 mM in 25 mM sodium phosphate buffer) at pH 8 for 30 min, to avoid oxidation and permit the posterior disulfide exchange. After, the reduced biocatalyst was washed with distilled water until the DTT smell disappears. Then 0.2 g of the reduced biocatalyst was added to 3 mL of 2-PDS solution (1.5 mM substrate in a mixture of DMSO (5% *v*/*v*) in 25mM phosphate buffer) at pH 8.0 for 1 h, and then the suspension was abundantly washed with distilled water. The full cysteine PDS activation was confirmed by spectrophotometric assay. Then, 1 mL of AcN-Cys(SH)-Phe-Gly-Phe-Gly-Phe-CONH_2_ (p) solution (0.2 mg/mL) was added to the 2 mL of PDS-GTL variant for 1 h. The peptide (p) was previously treated with a solution of sodium borohydride (1 mg/mL) in 250 mM phosphate at pH 10.0 for 30 min (to conserve the thiol group in a reductive form), and then pH was adjusted to 8.0 with diluted HCl solution to destroy the remaining sodium borohydride.

After that, the chemically modified GTL variant was incubated in 2 mL of 1.25% (*v*/*v*) sucrose monolaureate in 250 mM phosphate at pH 8.0 for 1 h, and the full desorption of the modified GTLσ-A193Cp variant was confirmed by activity assay and SDS electrophoresis. This solution had an approximate protein concentration of 0.9 mg/mL. 

### 3.5. Enzymatic Activity of the Artificial GTL Variants on Hydrolysis of p-Nitrophenyl Propionate (pNPP)

The activity of the GTL variants was analyzed spectrophotometrically measuring the increment in absorbance at 348 nm produced by the release of *p*-nitrophenol (pNP) (є = 5150 M^−1^ cm^−1^) in the hydrolysis of 0.4 mM pNPP in 25 mM sodium phosphate at pH 7 and 25 °C. To initialize the reaction, 0.05–0.2 mL of lipase solution or suspension was added to 2.5 mL of substrate solution.

### 3.6. Synthesis of GTLσ-A193Cp-PdNPs Bionanohybrid

Pd(OAc)_2_ (5mg) was dissolved in 1 mL of DMF and then added to 4 mL of GTLσ-A193Cp solution (the previously desorbed enzyme solution was diluted 1:1 with distilled water, obtaining 0.45 mg/mL solution) under vigorous magnetic stirring at 25 °C. The amount of solvent (20% *v*/*v*) was needed to ensure the full dissolution of the palladium salt in aqueous media. The solution was kept on gentle magnetic stirring for 24 h at room temperature. The resulting final suspension was separated by centrifugation (8000 rpm; 4 °C; 35 min), and the recovered pellet was washed two times with a 20% *v*/*v* DMF/distilled water solution for the removal of the remainders of palladium salt, and two more times with 5 mL of distilled water. After the last washing, the suspension was lyophilized to obtain the solid for characterization and use in the reaction. 

### 3.7. General Procedure for Heck Reaction

The reaction mixture was prepared in a screw-sealed glass vessel, containing 1 mL of solvent solution (either 1 mL of pure DMF or DMF/water), 0.274 mmol (30 µL) of iodobenzene and 0.55 mmol (59 µL) of ethylacrylate. This solution was preheated at 65 °C for 5 min under magnetic stirring for the complete solubilization of the substrates; then, 1 mg of palladium catalyst was added to the mixture (containing 0.4 mg of Pd) as well as 0.412 mmol of a base (either triethylamine, DMAP or DIPEA) and was kept under vigorous magnetic stirring at 65 °C for the indicated times. Samples (80 µL) were taken at different times, and the reaction was followed by HPLC. Samples were first centrifuged at 8000 rpm for 5 min, and then 10 µL was diluted 1000 times in a solution of acetonitrile:water 1:1. The reaction outgoing was monitored by HPLC analysis of the reaction samples at different times. The analysis conditions were performed with a Kromasil-C8 (150 × 4.6 mm and 5 µm Ø), at a flow of 1.0 mL/min; λ: 254 nm; and a mobile phase: 50% (*v*/*v*) ACN in MilliQ water. In these conditions, the retention times were: 4.18 min for ethyl acrylate (**2**), 11.1 min for (E)-trans ethyl cinnamate (**3**) and 13.25 min for iodobenzene (**1**). The E configuration was confirmed by HPLC using the (E) and (Z)-3 standards. Retention time for the (Z)-3 was 10.35 min (see Supplementary Materials).

### 3.8. Recyclability Test

The protocol for Heck reaction mentioned previously was followed, using triethylamine as a base and 75% (*v*/*v*) DMF in water as a solvent. After each cycle (stopping the reaction around 50% conversion), the reaction solution was centrifuged, and the nanobiohybrid was washed two times with DMF and left to dry for 15 min in a fume hood. This recyclability test was performed for five cycles.

## 4. Conclusions

Herein we have demonstrated the applicability of a new kind of Pd bionanohybrid in the Heck reaction.

A recombinant lipase from *Geobacillus thermocatenulatus* was selected, which was genetically modified by introducing a cysteine at position 193 and then this cysteine modified with a tailor-made peptide. This designed semisynthetic lipase was applied successfully to produce a heterogeneous bionanohybrid formed by ultra-small Pd nanoparticles embedded on the protein network. The applicability of a recombinant protein to produce the hybrid would permit to the accessibility of reproducible and multi-milligram scaled bionanohybrid. The use of a thermoalkalophilic enzyme in the formation of the nanobiohybrid is an advantage, which was demonstrated by this new hybrid catalyzing the Heck reaction in pure DMF at 65 °C, in comparison with other bionanohybrid using another lipase where no conversion was observed at these conditions [18]. The bionanohybrid was excellent using 10% water (*v*/*v*) as co-solvent in DMF and was quite stable being recyclable without losing the activity or *trans*-selectivity. 

Therefore, these results demonstrate that this could be an excellent candidate for application in organic synthesis, even in harsh conditions.

## Figures and Tables

**Table 1:** Heck coupling of iodobenzene with ethyl acrylate catalyzed by GTLσ-A193Cp-PdNPs bionanohybrid ^[a]^.

Entry	Base	Conversion of 3 [%]	TON	TOF (h^−1^)	Selectivity *trans*/*cis* (%)
1	DMAP	0	-	-	-
2	DMAP/NEt_3_	20	18.27	0.76	>99
3	NEt_3_	78(>99) ^[b]^	71.2(91.33)	2.96 (1.90)	>99
4	DIPEA	61	55.7	2.32	>99
5	NEt_3_	44 ^[c]^	40.2	1.67	>99
6	NEt_3_	7 ^[d]^	6.4	0.27	>99
7	NEt_3_	17 ^[e]^	15.52	0.64	>99

^[a]^ Conditions: **1** (0.274 mmol), **2** (0.55 mmol), 1 mL DMF/H_2_O (75/25), 1.5 equiv base, 1 mg of nanocatalyst, 24 h, 65 °C. ^[b]^ after 48 h. ^[c]^ 5 equiv base. ^[d]^ 10 equiv base. ^[e]^ 10 equiv **2.**

**Table 2:** Heck coupling of iodobenzene with ethyl acrylate catalyzed by GTLσ-A193Cp-PdNPs bionanohybrid as catalyst at different aqueous content. ^[a]^

Entry	Co-Solvent [% *v*/*v*, H_2_O]	T [°C]	Conversion of 3 [%]	TON	TOF (h^−1^)	Selectivity *trans*/*cis* (%)	Reference
1	0	65	56	51.15	2.13	>99	This work
2	10	65	86	78.54	3.27	>99	This work
3	25	65	78	71.23	2.96	>99	This work
4	40	65	50	45.65	1.90	>99	This work
5	0	70	0	-	-	-	[18]
7	50	70	20	18.27	0.76	>99	[18]

^[a]^ Conditions: **1** (0.274 mmol), **2** (0.55 mmol), 1 mL DMF, 1.5 equiv NEt_3_, 1 mg of nanocatalysts, 24 h, 65 °C.

## Supplementary Materials

The following are available online: Figure S1: Crystal structure of GTL native and modified together with the lid area marked key amino acids, Table S1: Suzuki-Miyaura cross coupling of bromobenzene (BB) with phenylboronic acid (PBA) catalyzed by bionanohybrid, Table S2: Suzuki-Miyaura cross coupling of bromobenzene (BB) with phenylboronic acid (PBA) catalyzed by bionanohybrid.
