# Supplementary material for: Ultra-Small Pd(0) Nanoparticles into a Designed Semisynthetic Lipase: An Efficient and Recyclable Heterogeneous Biohybrid Catalyst for the Heck Reaction under Mild Conditions
Source: Molecules. 2018 Sep 14;23(9):2358. doi: 10.3390/molecules23092358 (PMC6225421; doi:10.3390/molecules23092358)
Supplement: Supplementary file 1 [file molecules-23-02358-s001.pdf]

SUPPORTING INFORMATION

Ultra-small Pd(0) nanoparticles into a designed semisynthetic lipase: an efficient and recyclable heterogeneous biohybrid catalyst for the Heck reaction under mild conditions

David Lopez-Tejedor [1], Blanca de las Rivas [2] and Jose M. Palomo [1,*]

[1] Institute of Catalysis (CSIC), Department of Biocatalysis, Marie Curie 2, Cantoblanco Campus UAM, 28049, Fax: (+34)-91-585-4760

[2] Institute of Food Science, Technology and Nutrition (ICTAN-CSIC), José Antonio Novais 10, 28040 Madrid Phone + 34 91 549 2300, Fax. + 34 91 549 3627

* Correspondence: josempalomo@icp.csic.es; Tel.: +34-91-585-4768

Suzuki-Miyaura reaction between bromobenzene with phenylboronic acid catalyzed by Bionanohybrid. Bromobenzene (0.05 mmol) was added to a 1.5 mL screw-sealed vessel containing phenylboronic acid (0.055 mmol), NaOH (1.5 eq) and TBACl (0.0165 mmol) in distilled water: methanol (1:1) (1 mL). The mixture was kept at 50 °C under vigorous magnetic stirring for 5 min. After that, to initialize the reaction, the aqueous suspension of the GTLσ-A193Cp-PdNPs (1 mg; 0.4 mg of Pd) was added. The final suspension was left under vigorous magnetic stirring at 50°C for 24 h. The reaction outgoing was monitored by HPLC analysis of the reaction's samples withdrawn at different times. The analysis conditions were performed with a Kromasil-C4 (150 × 4.6 mm and 5 μm Ø), at a flow of 1.5 mL / min; λ: 254 nm; and a mobile phase: 50% (v/v) ACN in MilliQ water. The R_t of biaryl product was corroborated using standard products.

Sonogashira reaction between iodoanisole and propargyl alcohol catalyzed by Bionanohybrid. Iodoanisole (47 mg, 1 equiv) was added to a 3.5 mL screw-sealed vessel containing 15 µL of propargyl alcohol (1.3 equiv) and 2 mg of CuI (0.05 equiv.) in 1.5 mL of thiethylamine. The mixture was kept at room temperature under vigorous magnetic stirring for 5 min. After that, to initialize the reaction, GTLσ-A193Cp-PdNPs (7 mg, 3 mg of Pd) was added. The final suspension was left under vigorous magnetic stirring at room temperature for 24 h. The reaction outgoing was monitored by TLC. The R_t was corroborated are and agree with Belot, A. et al (*Angew. Chem. Int . Ed.* **2009**, *48* , 8923).

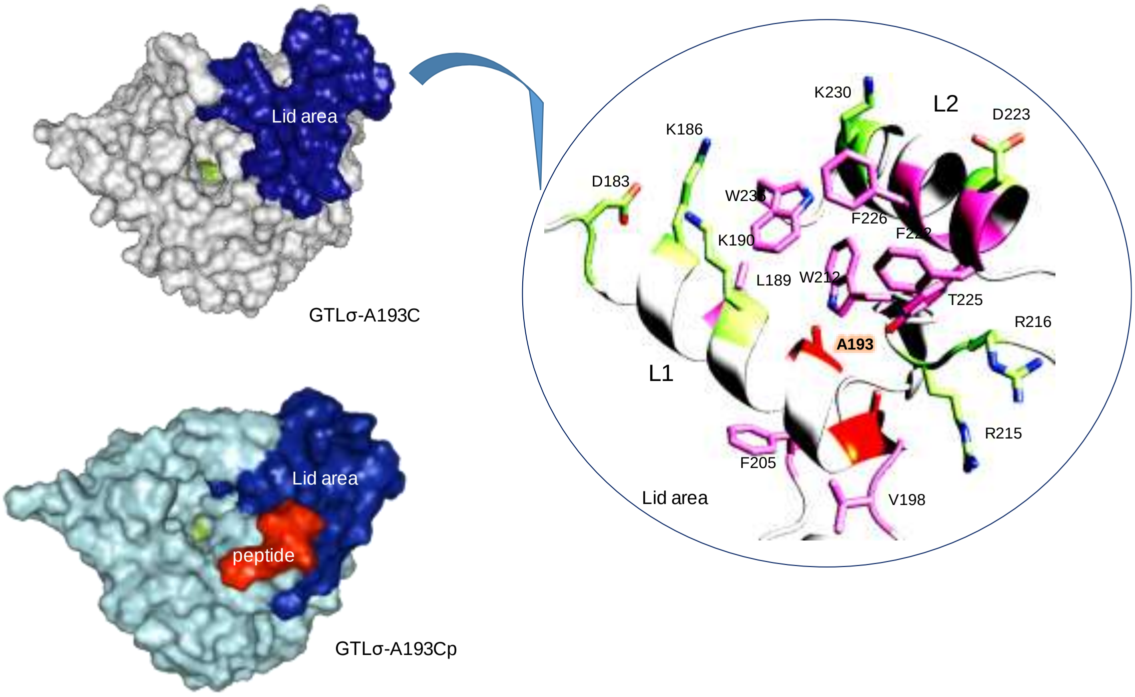

Figure S1. Crystal structure of GTL native and modified together with the lid area marked key amino acids. The structure was obtained from the PDB (ID: 2W22), and the picture was created by using PyMOL v. 0.99.

Table S1. Suzuki-Miyaura cross coupling of bromobenzene (BB) with phenylboronic acid (PBA) catalyzed by bionanohybrid.

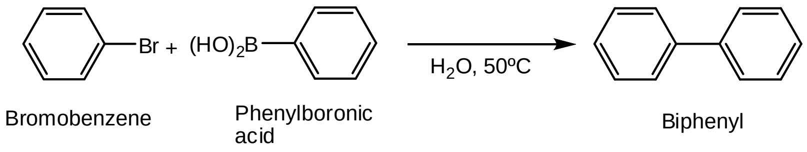

**Catalyst**	**Conversion (%)[a]**	**TON**	**TOF (h^{-1})**
GTLσ-A193Cp-PdNPs	62	10.33	0.43

[a]Reaction conditions: BB (0.05 mmol), PBA (0.055 mmol), H_2O/MeOH 50/50 (1 mL), TBACl(0.0165 mmol), 1 mg nanohybrid, 50ºC,24 h

Table S2. Suzuki-Miyaura cross coupling of bromobenzene (BB) with phenylboronic acid (PBA) catalyzed by bionanohybrid.

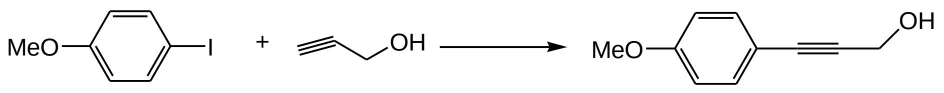

iodoanisole propargyl alcohol

Catalyst	Conversion (%)[b]
GTLσ-A193Cp-PdNPs	10

[a]Reaction conditions: Iodoanisole (47 mg, 1 equiv), propargyl alcohol (15 µL,1.3 equiv), CuI (0.2 mg, 0.05 equiv) , thiethylamine (1.5 mL), 7 mg nanohybrid, r.t.,24 h
